# Bioinspired artificial nanovesicles engineered from 3D spheroid-cultured UC-MSCs enhance angiogenic activity *in vitro*: functional proof-of-concept for ischemic applications

**DOI:** 10.3389/fcell.2026.1695559

**Published:** 2026-02-06

**Authors:** Prakash Gangadaran, Ramya Lakshmi Rajendran, Ji Min Oh, Jaeyoung Son, Chae Moon Hong, Byeong-Cheol Ahn

**Affiliations:** 1 Department of Nuclear Medicine, School of Medicine, Kyungpook National University, Daegu, Republic of Korea; 2 Cardiovascular Research Institute, Kyungpook National University, Daegu, Republic of Korea; 3 BK21 FOUR KNU Convergence Educational Program of Biomedical Sciences for Creative Future Talents, Department of Biomedical Sciences, School of Medicine, Kyungpook National University, Daegu, Republic of Korea; 4 Department of Nuclear Medicine, Kyungpook National University Hospital, Daegu, Republic of Korea

**Keywords:** angiogenesis, artificial nanovesicles, ischemia, mesenchymal stem cells, three-dimensional

## Abstract

Artificial nanovesicles (aNVs) derived from cells may mimic naturally secreted extracellular vesicles (EVs) and are becoming popular in biomedical research. We isolated aNVs from two-dimensional (2D)- and three-dimensional (3D)-cultured umbilical cord-derived mesenchymal stem cells (UC-MSCs) (aNVs^2D^ and aNVs^3D^, respectively) and characterized them using Western blotting and electron microscopy. The aNVs^3D^ showed higher expression of IL-6 and SDF-1α than aNVs^2D^. *In vitro* treatment with aNVs^2D^ and aNVs^3D^ resulted in their internalization into endothelial cells and the subsequent alteration of endothelial cell proliferation, migration, and tube formation. Both aNVs were positive for EVs and cell markers and were round in shape. Furthermore, aNVs^3D^ treatment enhanced endothelial cell proliferation, migration, and tube formation more effectively than aNVs^2D^ treatment. Our study demonstrates that aNVs3D are potent inducers of angiogenesis, indicating their potential in cell-free ischemia treatment.

## Graphical Abstract

**Figure:**
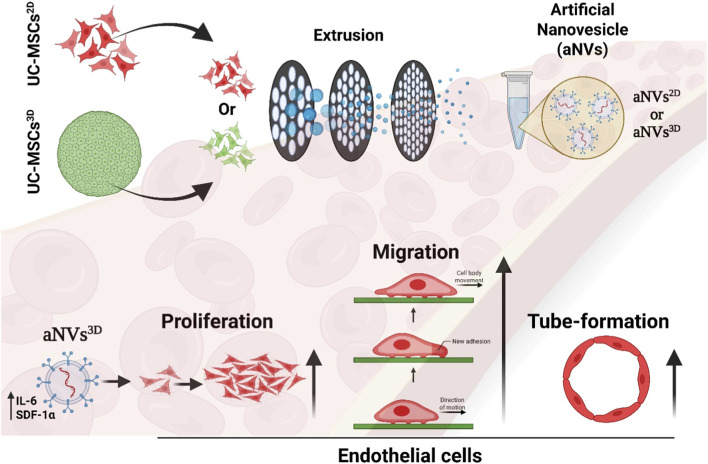

## 1 Introduction

Ischemic diseases, such as peripheral artery disease (PAD) and critical limb ischemia (CLI), pose significant clinical challenges by impairing blood flow, resulting in tissue necrosis and potential limb amputation (Ravi Mythili et al., 2025; Zemaitis et al., 2025). PAD is a widespread vascular condition that affects more than 200 million adults globally. The incidence of PAD has increased dramatically, reaching up to 20% in individuals aged ≥70 years (Zemaitis et al., 2025). Current therapeutic strategies, including drug administration (Arabzadeh et al., 2023), cell-based therapies (Desai et al., 2024; Huang et al., 2024), and surgical revascularization (Yuksel et al., 2018), have exhibited limited success in functional recovery. Hence, the development of a new and efficient therapeutic strategy is urgently required for effectively restoring vascularization in patients with ischemia.

Extracellular vesicles (EVs) are nanosized vesicles that are naturally secreted by almost all cells. They can carry and deliver bioactive biological materials, such as lipids, proteins, and nucleic acids (miRNA, mRNA, and DNA), to recipient cells and act as natural modulators in cell-to-cell communication (Rajendran et al., 2025a; Gangadaran et al., 2024); they can thus be used for treating diseases (Che Shaffi et al., 2024). EVs, particularly those derived from mesenchymal stem cells (MSCs), have emerged as promising candidates for regenerative therapies because of their ability to modulate angiogenesis, inflammation, and tissue regeneration (Samadi et al., 2025; Kumar et al., 2024). However, the clinical application of naturally secreted MSC-derived EVs is often hindered by their limited yield (Rajendran et al., 2025b). To overcome this limitation, engineered artificial nanovesicles (aNVs) have been developed as a bioinspired alternative that can mimic the functional properties of natural EVs with scalable production (Li et al., 2025; Lou et al., 2025; Li et al., 2024).

Recent studies suggest that the microenvironment in which MSCs are cultured significantly influences their secretory profile and therapeutic potential (Han et al., 2022; Xue et al., 2024). Although conventional two-dimensional (2D) culture systems have provided valuable insights into the biology and regenerative potential of MSCs, they fail to fully replicate the native three-dimensional (3D) microenvironment (Antoni et al., 2015; Yen et al., 2023). Notably, 3D spheroid culture conditions have been shown to enhance the paracrine effects of MSCs, enriching their secretome with proangiogenic factors (Han et al., 2022; Rajendran et al., 2024; Gangadaran et al., 2023). Therefore, we hypothesized that the 3D culture environment of cells enhances the production of angiogenic molecules, which could be transferred to engineered aNVs from 3D-cultured cells.

In this study, we assessed the potential of aNVs engineered from 3D spheroid-cultured umbilical cord-derived MSCs (UC-MSCs^3D^) (hereafter, aNVs^3D^) as novel proangiogenic therapeutic agents for promoting angiogenesis. We compared the therapeutic effects of engineered aNVs derived from 2D UC-MSC cultures (UC-MSCs^2D^) (hereafter, aNVs^2D^) and aNVs^3D^ on endothelial cells *in vitro*. Our findings provide valuable insights into the potential of aNVs^3D^ as a promising cell-free therapeutic strategy for ischemic diseases, paving the way for translational applications in regenerative medicine.

## 2 Materials and methods

### 2.1 Cells

Human UC-MSCs were purchased from the American Type Culture Collection (ATCC, Manassas, VA, United States) and cultured in the Mesenchymal Stem Cell Growth Kit-Low Serum for Adipose and Umbilical Cord-Derived MSCs (ATCC). Mouse endothelial cells (SVEC4-10EHR1) were purchased from ATCC and cultured in DMEM (Gibco, Carlsbad, CA, United States) supplemented with 10% FBS. All cells were maintained with 1% penicillin/streptomycin (Gibco) at 37 °C.

### 2.2 Monolayer 2D and 3D spheroid cultures

To generate UC-MSCs^2D^, UC-MSCs were cultured in flat bottom plates. To generate UC-MSCs^3D^, UC-MSCs (1X10^4^) were cultured in U-shaped 96-well plates as described previously (Rajendran et al., 2024). The morphology of UC-MSCs^3D^ was confirmed on day 5 by using a Nikon Eclipse Ti fluorescence microscope (Nikon Corp., Tokyo, Japan).

### 2.3 Engineering of aNVs

UC-MSCs^2D^ and UC-MSCs^3D^ were dissociated into single cells by gentle pipetting. Subsequently, aNVs were generated using a previously described method (Gopal et al., 2024). In brief, the cells were serially extruded five times through 10-μm, 5-μm, and 1-μm polycarbonate membrane filters (Nuclepore, Whatman, Inc., Clifton, NJ, United States) using a mini-extruder (Avanti Polar Lipids, Birmingham, AL, United States). The extruded sample was centrifuged at 4,000 × g for 10 min and subsequently filtered through a 0.45-μm filter. The samples were then ultracentrifuged at 100,000 × g for 1 h at 4 °C. The resuspended sample was further purified by performing differential gradient ultracentrifugation using OptiPrep density gradient medium (Iodixanol, Sigma-Aldrich, St. Louis, MO, United States) at 100,000 × g for 3 h at 4 °C. Following this, aNVs^2D^ and aNVs^3D^ were collected from the interface of the 60% and 20% iodixanol layers. The collected aNVs were then stored at −80 °C. The protein concentration in the aNVs was measured using the BCA Protein Assay Kit (Thermo Fisher Scientific, Waltham, MA, United States).

### 2.4 Western blotting

Western blotting was performed as described previously (Gopal et al., 2024). In brief, total proteins from the aNVs^2D^ and aNVs^3D^ were extracted in radioimmunoprecipitation assay (RIPA) buffer (Thermo Fisher Scientific, Waltham, MA, United States) and equally loaded of 10 µg protein per lane and separated using 10% sodium dodecyl sulfate–polyacrylamide gel electrophoresis. The samples were then transferred to polyvinylidene difluoride (PVDF) membranes (Millipore, Burlington, MA, United States). The membranes were blocked with 5% skim milk and probed with primary antibodies against ALIX, CD63, flotillin-1, and calnexin and then with a horseradish peroxidase-conjugated secondary antibody (Cell Signaling Technology, Danvers, MA, United States). After incubating the membrane with an enhanced chemiluminescence (ECL) solution (GE Healthcare Systems, Buckinghamshire, United Kingdom), the signals were observed using a chemiluminescence analyzer system (Vilber Lourmat, Marne-la-Vallée Cedex, France).

### 2.5 Transmission electron microscopy (TEM)

The aNVs^2D^ and aNVs^3D^ were visualized using TEM as described previously (Gangadaran et al., 2018). In brief, the aNVs^2D^ and aNVs^3D^ were resuspended in 2% paraformaldehyde for fixation. Subsequently, they were adsorbed onto Formvar–carbon-coated electron microscopy grids (Electron Microscopy Sciences, United States), air-dried, and fixed with 1% glutaraldehyde for 5 min at room temperature. The aNVs^2D^ and aNVs^3D^ on the grids were then negatively stained with 2% uranyl acetate, washed seven times with PBS, air-dried, and examined using TEM (HT 7700; Hitachi, Japan) at 100 kV.

### 2.6 Human cytokine array

Proteins extracted from aNVs^2D^ and aNVs^3D^ (50 µg per blot) were subjected to cytokine profiling using a Human Cytokine Array (RayBiotech, Peachtree Corners, GA, United States) according to the manufacturer’s instructions. Signal intensities were quantified using GelQuant.NET software (version 1.8.2; Biochem Lab Solutions, San Francisco, CA, United States) and represented in fold change after normalizing with positive control.

### 2.7 Internalization assay

The aNVs^2D^ and aNVs^3D^ were incubated with Vybrant™ DiD Cell-Labeling Solution (Thermo Fisher Scientific, Waltham, MA, United States) for 20 min. They were then reconstituted with PBS. The samples were processed in OptiPrep density gradient medium (Iodixanol, Sigma-Aldrich, St. Louis, MO, United States) at 100,000 × g for 3 h at 4 °C. The Dil-labeled aNVs^2D^ and aNVs^3D^ [aNVs^2D^ (DiD) and aNVs^3D^ (DiD), respectively] were collected from the interface as mentioned above. Endothelial cells (5 × 10^3^ cells/well) were cultured on eight-well slides and incubated overnight at 37 °C in 5% CO_2_. On the next day, 10 μg/mL of the aNVs^2D^, aNVs^3D^, aNVs^2D^ (DiD), and aNVs^3D^ (DiD) were incubated with endothelial cells for 2 h at 37 °C in 5% CO_2_. The slides were subsequently fixed with methanol and mounted using Vectashield antifade mounting medium with DAPI (Vector Laboratories, Burlingame, CA, United States). The internalization of aNVs^2D^ (DiD) and aNVs^3D^ (DiD) was observed under an AXIO microscope (Zeiss, Oberkochen, Baden-Württemberg, Germany).

### 2.8 Cell proliferation assay

Endothelial cells (2 × 10^4^ cells/well) were seeded in a 96-well plate and incubated overnight at 37 °C in 5% CO_2_. The cells were then treated with varying concentrations of aNVs^2D^ and aNVs^3D^ (0, 2.5, 5, 7.5, and 10 μg/mL) and incubated at 37 °C in 5% CO_2_. After 12 and 24 h, 10 μL of the Cell Counting Kit-8 (CCK-8; Dojindo, Kumamoto, Japan) reagent was added to each well and incubated for another 2 h at 37 °C in 5% CO_2_. Absorbance was then measured at 450 nm using a microplate reader, according to the manufacturer’s protocol.

### 2.9 Transwell migration assay

Transwell migration assays were performed in 24-well cell culture inserts containing an 8.0-µm-pore-size transparent PET membrane (BD Biosciences, Franklin Lakes, NJ, United States). Endothelial cells (5 × 10^4^ cells/insert) were plated in each upper insert with 0.5 mL of serum-free medium and treated with PBS (control), aNVs^2D^, or aNVs^3D^ (10 μg/mL each). Complete medium supplemented with 10% FBS was placed in the lower chamber and incubated at 37 °C in 5% CO_2_. After 12 and 24 h, the cells on the lower surface were fixed, stained with crystal violet, and imaged using a Nikon Eclipse Ti fluorescence microscope (Nikon Corp., Tokyo, Japan). The number of cells was counted in the captured field of images.

### 2.10 Tube formation assay

Matrigel Growth Factor Reduced (GFR) Basement Membrane Matrix (100% concentration) (Matrigel; Corning, Merck KGaA, Darmstadt, Germany) was coated onto 24-well plates and incubated at 37 °C in 5% CO_2_ for 1 h. Subsequently, 5 × 10^5^ endothelial cells were seeded onto the Matrigel. The endothelial cells were then treated with PBS (control), aNVs^2D^, or aNVs^3D^ (10 μg/mL each) and incubated at 37 °C in 5% CO_2_ at reduced FBS (2.5%) media. Tube formation ability was assessed after 3 h and 6 h under an AXIO microscope (Zeiss, Baden-Württemberg, Germany). The vessel percentage area, total vessel area, junction density, and total vessel length within the tubular structures were measured using AngioTool software (National Cancer Institute, Radiation Oncology Branch, Angiogenesis Core Facility, MD, United States).

### 2.11 Declaration of generative AI and AI-assisted technologies in the writing process

During the preparation of this work, the authors used Wordvice AI for proofreading and grammar checking. After using this tool/service, the authors reviewed and edited the content as needed and take full responsibility for the content of the published article.

### 2.12 Statistical analysis

All data are expressed as the means ± standard deviations (SDs). The differences between pairs of groups were statistically analyzed using Student’s t-test or Two-way ANOVA in Excel (Microsoft) or GraphPad Prism 10.6.1.892 (GraphPad Software Inc., San Diego, CA, United States). p < 0.05 was considered statistically significant.

## 3 Results

### 3.1 Establishment of UC-MSCs^2D^ and UC-MSCs^3D^

**FIGURE 1:**
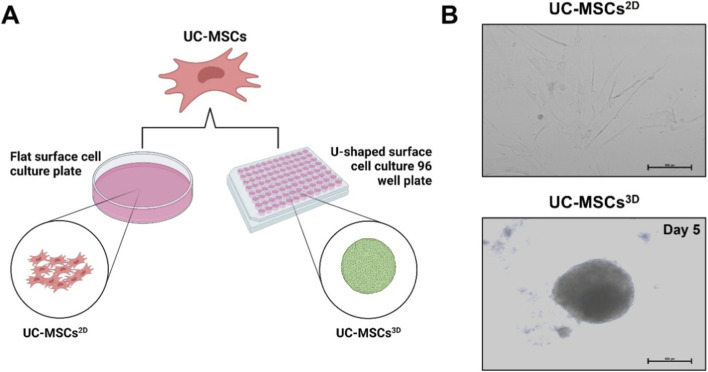
Establishment of 2D and 3D Cultures of Umbilical Cord-Derived Mesenchymal Stem Cells. **(A)** Schematic representation of 2D and 3D UC-MSC cultures, created with BioRender.com. **(B)** Images of 2D cultures of MSCs (UC-MSCs^2D^) in flat bottom plates and 3D cultures of MSCs (UC-MSCs^3D^) in U-shaped 96-well plates, with UC-MSCs^3D^ cultured up to day 5 (scale bar: 500 pixels).

Figure 1A illustrates the establishment of UC-MSCs^2D^ and UC-MSCs^3D^. As shown in the representative images, In the 2D cultures, the UC-MSCs adhered to the surface of the flat bottom plate and exhibited a characteristic spindle-shaped morphology. In contrast, after 5 days of culture in U-shaped 96-well plates, UC-MSCs^3D^ appeared as aggregated 3D spheres (Figure 1B). These results indicate the successful establishment of UC-MSCs^3D^.

### 3.2 Generation and characterization of aNVs^2D^ and aNVs^3D^

**FIGURE 2:**
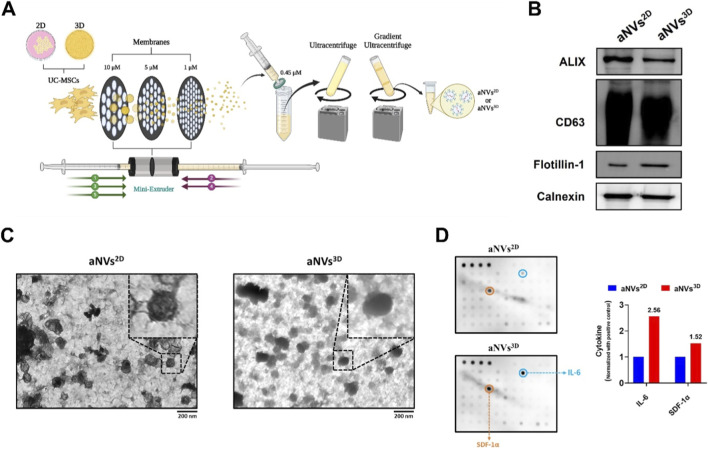
Generation and Characterization of aNVs^2D^ and aNVs^3D^. **(A)** Graphical illustration of the generation of aNVs^2D^ and aNVs^3D^ from UC-MSCs^2D^ and UC-MSCs^3D^, respectively, created with BioRender.com. **(B)** Western blot analysis of aNVs^2D^ and aNVs^3D^ (10 µg protein per lane) using antibodies against ALIX, CD63, flotillin-1, and calnexin. **(C)** TEM imaging of aNVs^2D^ and aNVs^3D^ (scale bar: 200 nm). **(D)** An array blot incubated with aNVs^2D^ and aNVs^3D^ lysate (50 µg per blot), the fold change of highly expressed cytokines was presented in graph.

UC-MSCs^2D^ and UC-MSCs^3D^ (dissociated by gentle pipetting) were extruded five times through membranes of various sizes (10, 5, and 1 µm), filtered, isolated, and purified to generate aNVs^2D^ and aNVs^3D^, respectively (Figure 2A). EVs and cell markers in both aNVs^2D^ and aNVs^3D^ were assessed using Western blotting. The analysis revealed that both aNVs^2D^ and aNVs^3D^ expressed the key EV markers ALIX, CD63 and flotillin-1 and the cellular marker calnexin (Figure 2B). TEM further revealed that both aNVs^2D^ and aNVs^3D^ exhibited a distinct spherical morphology (Figure 2C). Eighty human cytokines in aNVs^2D^ and aNVs^3D^ were analyzed by a human cytokine array, several cytokines were differently expressed in aNVs^2D^ and aNVs^3D^. Among the cytokines upregulated in aNVs^3D^, interleukin-6 (IL-6) and stromal cell–derived factor-1α (SDF-1α; CXCL12), are well-recognized regulators of angiogenesis (Figure 2D). These results indicate the successful generation and characterization of aNVs^2D^ and aNVs^3D^ and along with enrichment of angiogenic factors.

### 3.3 Internalization of aNVs^2D^ and aNVs^3D^ into endothelial cells

**FIGURE 3:**
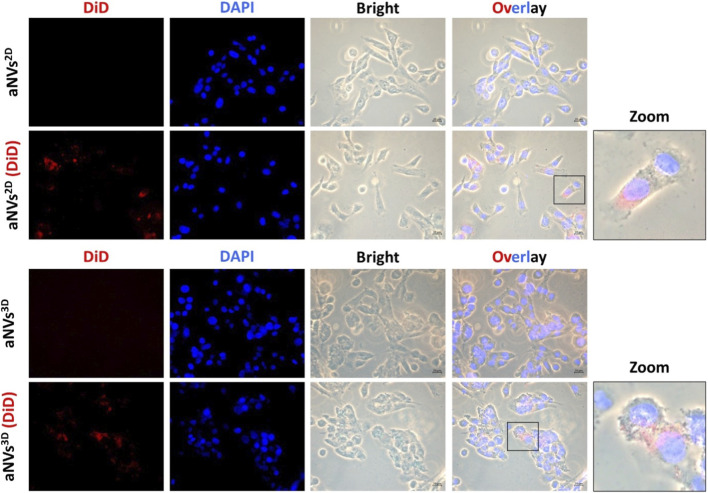
Internalization of aNVs^2D^ and aNVs^3D^ into Endothelial Cells. Fluorescence microscopy images demonstrate the internalization of aNVs^2D^ (DiD) and aNVs^3D^ (DiD) into endothelial cells (scale bar: 20 µm).

The internalization of aNVs is important for therapeutic purposes because it allows the cargo of aNVs to be delivered into recipient cells^8,22^. The active internalization of aNVs^2D^ and aNVs^3D^ was assessed using a fluorescence-based technique. In brief, aNVs^2D^ and aNVs^3D^ were labeled with the lipophilic dye DiD, and unlabeled aNVs^2D^, unlabeled aNVs^3D^, aNVs^2D^ (DiD), and aNVs^3D^ (DiD) were then incubated with endothelial cells. Fluorescence microscopy images demonstrated that both aNVs^2D^ (DiD) and aNVs^3D^ (DiD) were actively internalized into endothelial cells. This internalization was predominantly observed within the cytoplasm. No signals were observed in unlabeled aNVs^2D^ and aNVs^3D^ incubated with endothelial cells (Figure 3). These results indicate that both aNVs^2D^ (DiD) and aNVs^3D^ (DiD) targeted and were internalized into endothelial cells.

### 3.4 aNVs^3D^ accelerated the proliferation and migration of endothelial cells

**FIGURE 4:**
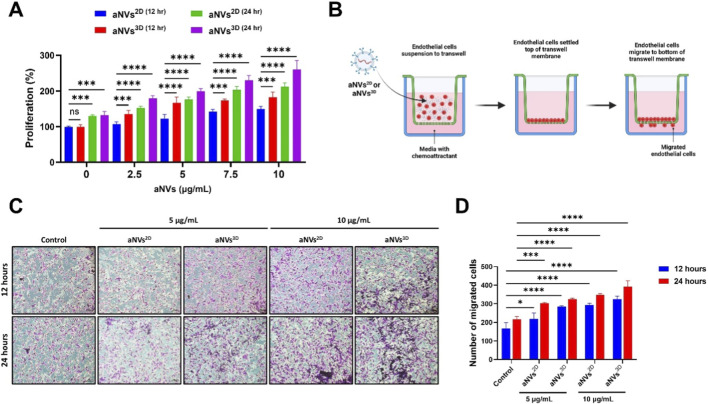
aNVs^3D^ Promoted the Proliferation and Migration of Endothelial Cells. **(A)** Endothelial cells were treated with aNVs^2D^ and aNVs^3D^ (0, 2.5, 5, and 10 μg/mL) for 12 h and 24 h, and cell proliferation (n = 4) was assessed using the CCK-8 assay. **(B)** Illustration of the experiment to assess endothelial cell migration following treatment with aNVs, created with BioRender.com. **(C)** Representative image of transwell migration of endothelial cells treated with aNVs^2D^ and aNVs^3D^ (5 and 10 μg/mL) at 12 h and 24 h (scale bar: 100 µm). **(D)** Quantitative analysis of the transwell migration assay presented in **(C)** (n = 3). Statistical significance is denoted as follows: *p < 0.05; **p < 0.01, ***p < 0.001 and ****p < 0.0001, as determined by Two-way ANOVA.

Endothelial cells were treated with aNVs^2D^ and aNVs^3D^ (0, 2.5, 5, 7.5, and 10 μg/mL) for 12 h and 24 h, and their proliferation was assessed using the CCK-8 assay. Cell proliferation was enhanced on treatment with aNVs^2D^ and aNVs^3D^. Treatment with aNVs^3D^ increased endothelial cell proliferation significantly more effectively than treatment with aNVs^2D^ at all tested concentrations and two time points (two-way ANOVA, p < 0.001 to p < 0.0001). (Figure 4A). The migration of endothelial cells is crucial for sprouting, which results in blood vessel formation. Therefore, endothelial cell migration was performed as illustrated in Figure 4B. The transwell migration assay demonstrated that both aNVs^2D^ and aNVs^3D^ (5 and 10 μg/mL) enhanced endothelial cell migration at 12 h and 24 h compared to the control across all tested concentrations and two time points (two-way ANOVA, p < 0.001 to p < 0.0001). Notably, aNVs^3D^ induced a higher migration rate than aNVs^3D^ (Figures 4C,D).

### 3.5 aNVs^3D^ promoted vessel-like tube formation *in vitro*

**FIGURE 5:**
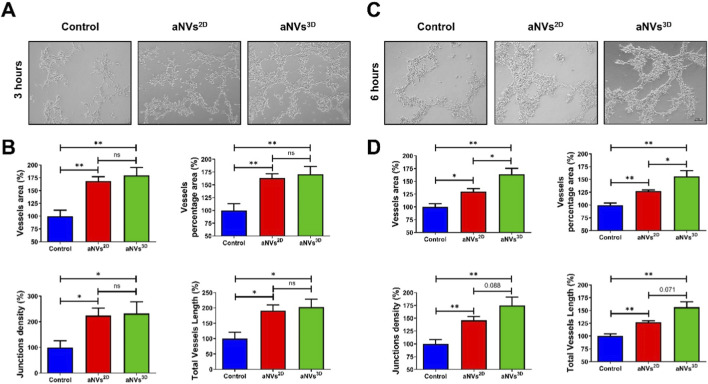
aNVs^3D^ Promoted Vessel-Like Tube Formation *In Vitro.* **(A,B)** Representative microscopic images showing vessel-like tube formation by endothelial cells treated with aNVs^2D^ and aNVs^3D^ in Matrigel (scale bar: 100 µm) at 3 h and 6 h **(C,D)** Quantification of % of vessel percentage area total vessel area, junctions density, and total vessels length analyzed using AngioTool for **(A)** (n = 3). Statistical significance determined by Student’s t-test is denoted as follows. ns: no significance, *p: <0.05, and **p: <0.01.

The effect of aNVs^3D^ on *in vitro* capillary-like network formation was assessed using a tube formation assay in Matrigel. Endothelial cells were treated with aNVs^2D^ and aNVs^3D^ for 3 and 6 h. Subsequently, microscopic images were taken for visualizing vessel-like tube formation (Figures 5A,C). The images were assessed using AngioTool. Quantitative results at 3 h revealed that treatment with aNVs^3D^ promoted the vessel percentage area, total vessel area, junction density, and total vessel length compared to treatment with aNVs^2D^ and the control (p < 0.05 or p < 0.01) (Figure 5B). At 6 h, Quantitative assessment showed that both aNVs^2D^ and aNVs^3D^ significantly enhanced vessel area, vessel percentage area, junction density, and total vessel length relative to the control (p < 0.05 or p < 0.01). Importantly, aNVs^3D^ induced significantly greater vessel area and vessel percentage area compared with aNVs^2D^ (*p < 0.05), suggesting superior angiogenic efficacy. While junction density and total vessel length were also higher in the aNVs^3D^ group than in the aNVs^2D^ group, these differences showed a strong increasing trend but did not reach statistical significance (p = 0.088 and p = 0.071, respectively) (Figure 5D). These findings suggest that treatment with aNVs^3D^ is more effective in promoting capillary-like network formation than treatment with aNVs^2D^.

## 4 Discussion

Stem cells have garnered significant attention as a promising new treatment for PAD and CLI (Samura et al., 2017). Most of the therapeutic effects of MSCs are attributed to secretory factors, including EVs. Most studies have utilized natural MSC-derived EVs for the treatment of ischemic diseases (Gregorius et al., 2021; Gangadaran et al., 2021; Gangadaran et al., 2017). However, achieving therapeutic outcomes from natural EVs is a lengthy process that requires the collection of large volumes of conditioned media and extensive cell expansion (Fan et al., 2020; Hao et al., 2021). In this study, aNVs^3D^ were created to address the limitations of natural EVs derived from 2D cells that are currently used in research on angiogenesis and ischemic diseases. The rationale for employing 3D spheroid culture is its capacity to more accurately replicate the native stem cell microenvironment, thereby enhancing cellular interactions and the secretion of therapeutic factors (Yen et al., 2023; Gao et al., 2024; Lu et al., 2024).

The 3D cell culture model can be developed using either scaffold-free methods, in which cells aggregate without an extracellular matrix (ECM), or scaffold-based approaches, in which cell growth occurs within an ECM environment (Lee et al., 2023). In this study, we used scaffold-free methods and generated UC-MSCs^3D^ in a U-shaped 96-well plate. Unlike other methods, this method facilitates easy collection of spheroids with uniform size (Chaicharoenaudomrung et al., 2019; Kim et al., 2024). In our study, the 3D spheroids showed consistent uniform size on day 5, indicating that each spheroid was cultured under the same culture condition and was likely to have the same biological characteristics, as reported previously (Lee et al., 2023). Hence, the generation of aNVs from uniform 3D spheroid-derived cells yields a more homogeneous therapeutic outcome than that from heterogeneous 3D spheroid cells.

aNVs^2D^ and aNVs^3D^ were engineered from UC-MSCs^2D^ and UC-MSCs^3D^, respectively, by serial extrusion with 10, 5, and 1 µM polycarbonate membranes, which NVs form via the self-assembly of lipid bilayer pieces from fragmented cells into vesicles, and were further purified through filtration and gradient ultracentrifugation. Our Western blot analysis revealed that both aNVs^2D^ and aNVs^3D^ were positive for EV markers, such as ALIX, CD63 and flotillin-1 (Hessvik and Llorente, 2017), indicating that aNVs are made of cell membranes, such as EVs. In addition, both aNVs^2D^ and aNVs^3D^ were positive for calnexin, an endoplasmic reticulum marker that is considered a negative marker for natural EVs. While natural EVs may only carry some cellular content, aNVs can theoretically encapsulate the entire cellular content (Sun et al., 2022). Both aNVs^2D^ and aNVs^3D^ were confirmed to be round in shape, consistent with the findings of previous studies on the generation of aNVs (Jang et al., 2013; Li et al., 2022). aNVs^3D^ exhibited enhanced angiogenic factors (Cohen et al., 1996; Ababneh et al., 2025) such as IL-6 and SDF-1α (CXCL12) compared with aNVs^2D^, this may enhance angiogenic effect of aNVs^3D^ as both are key pro-angiogenic cytokines. Consistent with our findings, previous studies have demonstrated that 3D culture enhanced the pro-angiogenic factors in cells, secretory factors including EVs (Ababneh et al., 2025; Rovere et al., 2023; Gangadaran et al., 2023).

The internalization of aNVs into cells is crucial for their therapeutic potential, enabling the delivery of functional biological materials and the activation of intracellular signaling pathways (Li et al., 2025). In our study, aNVs^2D^ and aNVs^3D^ actively interacted with endothelial cells and were internalized into them. During angiogenesis, i.e., the process of forming new blood vessels, endothelial cell proliferation (growth and division) is crucial for the expansion of the existing vascular network (Lorenc et al., 2024). We assessed whether internalization causes any changes in endothelial cell proliferation. Treatments with aNVs^2D^ and aNVs^3D^ augmented the proliferation of endothelial cells, with aNVs^3D^ inducing higher endothelial cell proliferation than aNVs^2D^. The migration of endothelial cells is the next step in the formation of new blood vessels (Michaelis, 2014). Treatment with aNVs^3D^ enhanced the migration of endothelial cells more effectively than treatment with aNVs^2D^. Our results revealed that aNVs^3D^ exhibited enhanced angiogenic effects, proving that 3D culture enhanced the angiogenic factors in cells that were eventually transferred to aNVs^3D^. Consistent with our findings, previous study has demonstrated that 3D MSC-derived EVs exhibit superior pro-proliferative enhanced migration, and improved tube formation effects on endothelial cell compared with their 2D-derived EVs (Gao et al., 2021) and another study demonstrated that a 3D culture system significantly enhanced angiogenic therapeutic efficacy, showing improved pro-angiogenic activity in endothelial cells *in vitro* as well as superior functional recovery in an *in vivo* heart repair model (Sun et al., 2023). Hence, we hypothesized that aNVs^3D^ can stimulate angiogenesis by inducing tube formation in endothelial cells. Compared to aNVs^2D^, aNVs^3D^ significantly improved tube formation in endothelial cells under Matrigel conditions. Overall, our results suggest that UC-MSCs^3D^ exhibit enhanced expression of proangiogenic factors, which are subsequently transferred to aNVs^3D^ during their generation. However, our study has some limitations. Although *in vitro* data strongly support the angiogenic efficacy of aNVs^3D^, their *in vivo* performance in promoting neovascularization in ischemic tissues still needs to be assessed. Furthermore, comprehensive molecular profiling of the vesicle cargo (e.g., proteomics and transcriptomics) could offer deeper insights into the mechanistic basis of the observed effects.

In conclusion, our findings highlight that aNVs^3D^ exhibit superior proangiogenic effects, representing a potent, scalable, and cell-free therapeutic strategy for ischemic diseases.

## Data Availability

The original contributions presented in the study are included in the article/supplementary material, further inquiries can be directed to the corresponding authors.
